# Duplication of the Antistasin-Like Structure Resulted in a New Anticoagulant Protein in the Medicinal Leech

**DOI:** 10.3390/biom16010155

**Published:** 2026-01-15

**Authors:** Ksenia A. Brovina, Vladislav V. Babenko, Valentin A. Manuvera, Pavel A. Bobrovsky, Daria D. Kharlampieva, Vassili N. Lazarev

**Affiliations:** 1 Center for Genetic Reprogramming and Gene Therapy, Lopukhin Federal Research and Clinical Center of Physical-Chemical Medicine of Federal Medical Biological Agency, Malaya Pirogovskaya, 1a, 119435 Moscow, Russia; pbobrovskiy@gmail.com (P.A.B.); lazarev@rcpcm.org (V.N.L.); 2 Moscow Center for Advanced Studies, 20, Kulakova Str., 123592 Moscow, Russia; 3 Lopukhin Federal Research and Clinical Center of Physical-Chemical Medicine of Federal Medical Biological Agency, Malaya Pirogovskaya, 1a, 119435 Moscow, Russia; daniorerio34@gmail.com (V.V.B.); harlampieva_d@mail.ru (D.D.K.)

**Keywords:** anticoagulant, clotting test, *Hirudo medicinalis*, leech, recombinant protein

## Abstract

Blood-sucking organisms produce various anticoagulant proteins that prevent blood clotting in their prey. Even in well-studied species like *Hirudo medicinalis*, many such proteins remain unidentified. We previously described a novel cysteine-rich anticoagulant (CRA), a distant homolog of antistasin. Later, we discovered another, much larger homolog in the medicinal leech. Its amino acid sequence is also highly cysteine-rich. Analysis of cysteine patterns showed four antistasin-like domain motifs, with one of them strongly disrupted. Since both antistasin and CRA contain two such domains, the new protein represents a duplicated antistasin-like structure. We cloned its cDNA, expressed the recombinant protein in *Escherichia coli*, purified it by metal-chelate chromatography, refolded it, and tested its anticoagulant properties. Using standard clinical assays—activated partial thromboplastin time, prothrombin time, and thrombin time—we found that the protein inhibited coagulation in all tests, though to varying degrees. These findings suggest that different antistasin-like anticoagulants in the leech enable it to block both intrinsic and extrinsic coagulation pathways, while hirudin inhibits the final step of clot formation. The combination of different anticoagulant proteins allows the leech to effectively prevent the prey’s blood from clotting during feeding.

## 1. Introduction

During evolution, blood-feeding leeches have acquired a number of features that facilitate hematophagy. Among these, they produce anticoagulants that enter the host’s bloodstream with saliva during feeding. The best-known member of this group is hirudin, a potent thrombin inhibitor discovered more than a century ago and characterized in detail in subsequent biochemical studies [1]. However, leech saliva contains a diverse set of anticoagulant and antiplatelet factors, and the full spectrum of these molecules remains far from completely explored. A typical mechanism by which leeches disrupt hemostasis is the selective inhibition of serine proteases in the coagulation cascade [2,3]. For instance, hirudin inhibits thrombin, preventing the conversion of fibrinogen to fibrin and the formation of a primary fibrin network. The protein antistasin (ANS) from the Mexican leech, which inhibits factor Xa [4], also belongs to this group of protease inhibitors [5]. ANS and its homologs were described in American leeches at the end of the last century [6,7]. In Europe, the most well-known medicinal leeches (*H. medicinalis, H. verbana, H. orientalis*) possess only shortened homologs composed of a single domain instead of two, namely hirustasin [8] and bdellastasin [9], which lack anticoagulant activity. Only the European duck leech *Theromyzon tessulatum* has a single-domain homolog of antistasin named therostasin, which inhibits factor Xa [10]. Recently, interest in identifying novel leech proteins affecting human hemostasis has grown substantially. Most contemporary studies rely heavily on transcriptomic analyses, typically searching for homologs of known anticoagulants [2,11,12,13,14]. However, this strategy inherently limits discovery: entire classes of novel proteins remain undetected simply because they are not represented in existing databases. Expanding search strategies beyond strict homology-based approaches has therefore become crucial for uncovering previously unrecognized anticoagulants in leeches. Previously, we used such a homology-based search for known anticoagulants (NCBI BioProject PRJNA257563), particularly antistasins, which led to the identification of the protein with robust anticoagulant activity—the cysteine-rich anticoagulant (CRA) [15,16]. CRA exhibits a cysteine distribution pattern similar to that of ANS, which is repeated twice and corresponds to the two structural domains of ANS [17]. Outside of this pattern, homology between the amino acid sequences of CRA and ANS is virtually absent. This likely explains why anticoagulants related to ANS had not been described in European leeches for a long time. A significant difference in CRA is the presence of a C-terminal motif, the removal of which leads to a loss of the protein’s affinity for membrane vesicles and alters its specificity in anticoagulant tests. Furthermore, the two domains of CRA are connected by a shorter and more rigid linker than the domains of ANS [16]. Later, genes encoding proteins of similar domain architecture were described in the leech *Hirudinaria bpling* [18], and they are very likely present in other leech species as well. In the study by Liu et al. [14], two antistasin genes were identified, and the corresponding protein sequences were found to be relatively conserved. However, the sequence alignment shown in Figure 4 [14] reveals a group of cysteine-rich anticoagulant proteins that, in addition to the two cysteine patterns characteristic of antistasins, also possess a C-terminal motif typical for CRA.

After we obtained recombinant CRA and discovered its ability to inhibit blood coagulation, we decided to perform a renewed search of the *H. medicinalis* genomic and transcriptomic datasets to identify previously uncharacterized anticoagulant proteins. This time, however, we used CRA, rather than ANS, as the reference molecule. As a result, we discovered another gene that appears to encode a duplicated paralog of CRA, consisting of four domains instead of two. The gene is located in assembly ASM1180080v1 in scaffold 418. Gene (g6902.t1) contains 13 exons; the annotated sequence is included in the supplementary data to the article [19]. The corresponding transcript is deposited in GeneBank ID PX804116. We cloned the cDNA and produced the recombinant protein, which we named MCRA (Multiple-CRA). This protein, like CRA, demonstrated high activity in laboratory coagulation assays. In this short communication, we report the identification, recombinant production, and preliminary functional assessment of MCRA protein.

## 2. Materials and Methods

### 2.1. The Construction of the Expression Plasmid

The cDNA from *H. medicinalis* salivary gland cells was obtained from laser-microdissected material and used in this study as the matrix. PCR amplification with oligonucleotides 5′-GTGGTGGTGCTCGAGTTTTCCAAATTTCTTCTTGAACCA-3′ (forward) and 5′-GGAGATATACATATGGTACATCCTTATTTTGGCGACT-3′ (reverse) was utilized to obtain a DNA fragment encoding the mature MCRA protein (signal peptide was excluded). The expression plasmid was obtained by the PIPE method [20]. For this, the plasmid pET-22b(+) (Novagen, Darmstadt, Germany) was amplified by PCR without the region encoding the pelB signal peptide. The oligonucleotides PETPIPE-1N (5′-CATATGTATATCTCCTTCTTAAAGTTAAAC-3′) and PETPIPE-2N (5′-CTCGAGCACCACCACCACCACCACTGAGATC-3′) were used. The resulting amplicons were mixed in a 5:1 molar ratio. Next, the mixture was used to transform *Escherichia coli* TOP10 cells (Invitrogen, Thermo Fisher Scientific, Waltham, MA, USA). Colonies carrying recombinant plasmids were identified by PCR. Subsequently, the plasmid pET-hmMCRA was isolated from the selected colonies. The structure of the target sequence was verified by automatic Sanger sequencing. The recombinant insert in plasmid DNA isolated from ten individual clones was sequenced. Unique substitutions present in only a single sequence were excluded as likely experimental artifacts arising during amplification. The plasmid map is provided in Supplementary Materials (Figure S1). The cDNA sequence is provided in Supplementary Materials (Text S1) and in GenBank (ID PX804116). The pET-hmCRA plasmid we constructed in a similar way earlier [16].

### 2.2. Protein Expression and Purification

*E. coli* BL21(DE3) gold cells (Novagen, Madison, WI, USA) were transformed with the pET-hmMCRA or pET-hmCRA plasmid. Transformants were cultivated in a shaker at 37 °C for six hours and further used to inoculate 250 mL of autoinduction TB medium (24 g/L yeast extract, 12 g/L tryptone, 4 g/L glycerol, 0.17 M KH_2_PO_4_, 0.72 M K_2_HPO_4_, 150 mg/L ampicillin, 0.5 g/L glucose, 1 g/L lactose). Bacterial cultures were incubated in 1 L flasks for 18 h at 37 °C under constant agitation (220 rpm) and subsequently harvested by centrifugation.

The same isolation and refolding protocol was applied to both proteins, CRA and MCRA. The cell pellet was resuspended in US buffer (PBS, 0.1% Triton X-100, 0.1 g/L lysozyme, 5 mM EDTA). Then, chilled cell suspension was disrupted by sonication using a Branson 250 Sonifier (Branson Ultrasonics, Danbury, CT, USA) at 22 kHz for 10 min. The solution was clarified by centrifugation, then the pellet was resuspended in 2% Triton X-100 using a sonifier and centrifuged. The washed pellet was solubilized in buffer AM (8 M urea, 20 mM Tris-Cl, 500 mM NaCl, 10 mM imidazole, pH 7.5). The column containing 10 mL of Ni Sepharose Fast Flow (GE Healthcare Bio-Sciences Corp., Marlborough, MA, USA) was equilibrated with buffer AM. After removal of the insoluble residue by centrifugation, the supernatant was applied to the column. Then, the column was washed with buffer AM and eluted with buffer EM (8 M urea, 20 mM Tris-Cl, 500 mM NaCl, 500 mM imidazole, pH 7.5).

The proteins were renatured by rapid 20-fold dilution in 20 mM Tris-Cl, pH 7.5, and further stored at 4 °C. For purification, the refolded protein solution was loaded onto a DEAE Sepharose FF column (4.5 mL, GE Healthcare Bio-Sciences Corp., Marlborough, MA, USA). The column was pre-equilibrated with 20 mM Tris-Cl, pH 7.5. Following sample application, the column was washed with 10 mL of the same buffer. The proteins were eluted using a 20 mL linear NaCl gradient from 20 mM Tris-Cl, pH 7.5, to 20 mM Tris-Cl, 1 M NaCl, pH 7.5. Chromatography was performed using an NGC chromatograph (Bio-Rad, Hercules, CA, USA). The fractions containing target proteins were identified by SDS-PAGE analysis with Coomassie G-250 staining. The fractions containing MCRA or CRA were then combined, re-analyzed by SDS-PAGE (Figure S2) and subsequently stored at −20 °C.

### 2.3. Protein Concentration Determination

The concentration of the purified proteins was measured based on absorbance at 280 nm using a Shimadzu UV-1900 spectrophotometer (Shimadzu Corp., Kyoto, Japan). The calculated molar and mass extinction coefficients are presented in Table 1.

### 2.4. Clotting Assays

All measurements were performed using APG4-03-Ph (EMCO LLC, Moscow, Russia) hematology analyzer and control plasma (Renam, Moscow, Russia). To assess protein anticoagulation activity, standard coagulation assays were used: activated partial thromboplastin time (aPTT), prothrombin time (PT), and thrombin time (TT), as described previously [21]. Each sample was measured in four replicates in parallel using four coagulometer measurement cells. A solution of medium-molecular-weight unfractionated sodium heparin (Hep) (Synthesis, Moscow, Russia) was used as positive control. Concentrations of the anticoagulant proteins are reported for the initial sample. In the reaction mixture, these concentrations are reduced sixfold for aPTT, fourfold for TT, and threefold for PT. For heparin, the total amount of IU per reaction is reported.

## 3. Results

### 3.1. Analysis of the Protein Sequence and Structure

**Figure 1:**
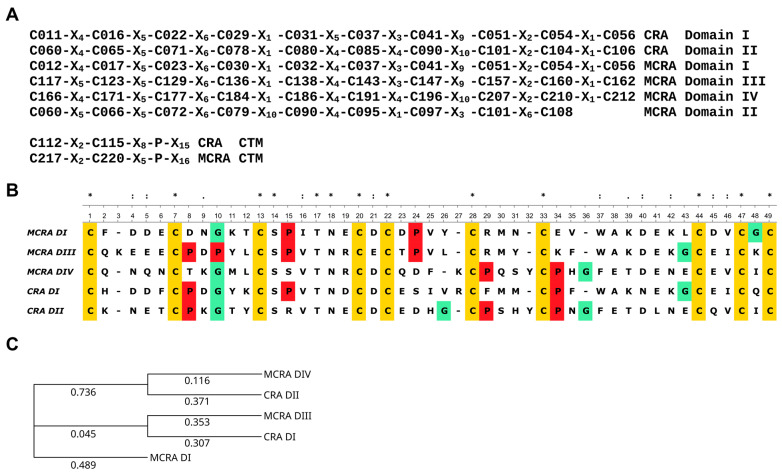
(**A**) Cysteine residue distribution patterns in the domains of CRA and MCRA. The letter ‘C’ with a number denotes the sequential position of the cysteine residue in the polypeptide chain; the letter ‘X’ with a number denotes the number of amino acid residues between adjacent cysteine residues. (**B**) Alignment of the amino acid sequences of the two domains of CRA and domains I, III, and IV of MCRA. Cysteine residues are highlighted in yellow, proline in red, and glycine in green as amino acid residues strongly influencing protein structure. Above the alignment, the symbol ‘*’ denotes conserved positions, the symbol ‘:’ denotes positions with residues of similar properties, and the symbol ‘.’ denotes positions with dissimilar residues. Sequence analysis and alignment were carried out via the Unipro UGENE platform [22]. (**C**) Phylogenetic tree of the two CRA domains and the MCRA domains (I, III, IV), constructed using the Neighbor-Joining method. Evolutionary distances were calculated using the Jones–Taylor–Thornton (JTT) model [23]. The numbers correspond to evolutionary distances.

The mature MCRA protein, excluding the signal peptide, consists of 242 amino acid residues, and 41 of them are cysteine residues (17% of total amount). MCRA has a calculated molecular mass of 28 kDa. The partial cDNA sequence and the amino acid sequence of the mature protein without the signal peptide are provided in Supplementary Materials (Texts S1 and S2). The very high cysteine content (16% of the 139 amino acid residues) is also a characteristic feature of CRA. Furthermore, the C-X_4_-C-X_5_-C-X_4,6_-C-X-C-X_4,5_-C-X_3,4_-C-X_9,10_-C-X_2,3_-C-X pattern, which is a feature of ANS, can be easily detected in both proteins. This pattern occurs twice in CRA and ANS and three times in MCRA, with one additional region possibly representing a modified version of it (Figure 1).

Both domains of CRA and domains I, III, and IV of MCRA are similar to each other, not only in the distribution of cysteine residues but also in a number of other positions. This allows us to propose a common ancestral origin. ANS also has two of these domains. Another protein from *H. medicinalis*, hirustasin, shares a similar structure [8]. However, although it is a protease inhibitor, it does not affect coagulation. In our opinion, the second domain of MCRA has the same origin. As seen in Figure 1A, the cysteine pattern is conserved with other domains up to Cys79, but then divergence occurs. This could be a consequence of recombination events during the union of two ancestral structures or other reasons. The phylogenetic tree of individual domains (Figure 1C) shows that domains I and III of MCRA and domain I of CRA form one group, while domain IV of MCRA and domain II of CRA form a second group. Based on this, it is quite reasonable to assume that CRA and MCRA did not originate independently from a single-domain structure. Rather, an initial duplication occurred, resulting in the development of a shared two-domain structure. This form then evolved to CRA, and a later duplication resulted in the appearance of MCRA. It should also be noted that both proteins have an extended C-terminal motif (CTM), which is absent in ANS and its homologs. In both CRA and MCRA proteins, the base of the CTM contains a C-X_2_-C motif and a proline residue located some distance away (Figure 1A). In the case of CRA, based on structure modeling, we previously suggested that this structure serves to create a specific orientation of the CTM relative to the protein molecule [16].

As one of the reviewers kindly pointed out, the MCRA DII region may represent a structure composed of duplicated first subdomains. The subdivision of the antistasin domain into two subdomains was described in the first and, apparently, still the only study reporting the X-ray structure of antistasin [17]. This hypothesis is highly attractive but cannot be accepted unambiguously. The three-dimensional structure of antistasin (PDB ID: 1SKZ) reveals two clearly separated folding units that correspond well to the first and second domains. Further subdivision of each domain into subdomains, as shown in Figure 5 of [17], is rather debatable. The two proposed subdomains are in close contact and are linked by a disulfide bond located in the central regions of each subdomain. It therefore seems questionable that each subdomain could fold independently of the other. Rather, both subdomains appear to form a single fold and thus constitute a single structural domain.

We extracted the putative subdomain sequences from the second domain of MCRA and compared them with the structures of the first subdomains of the other MCRA domains as well as CRA (Figure S2). The alignment shows that the N-terminal part of MCRA DII indeed resembles the first subdomain in terms of cysteine distribution. However, the serine residue at position 14 of the alignment, which is invariant in the other sequences, is absent. In contrast, the second putative subdomain exhibits a markedly different cysteine pattern and divergence at several other positions, although it contains a proline at position 8, which is present in three of the five full-length CRA and MCRA domains. In principle, such differences could have arisen from duplication of the first subdomain of MCRA DII followed by subsequent divergence. Alternatively, they could result from recombination errors. On the other hand, it is also possible that this region is unrelated to the first subdomain and instead originated from the second subdomain by similar mechanisms or represents a different type of sequence altogether. The available data are clearly insufficient to draw a definitive conclusion. Future analysis of MCRA orthologs may help to resolve this issue.

Structural modeling of MCRA using AlphaFold3 [24] is in good agreement with the conclusions drawn from the primary sequence analysis (Figure S3). Three antistasin-like domains (DI, DIII, and DIV) are clearly discernible. In contrast, the second domain (DII) adopts a different structural organization, and, as expected, the confidence level of its structural prediction is low (partly pLDDT < 50). A helical C-terminal motif (CTM) is also clearly identified, fully analogous to that of CRA [16].

### 3.2. Investigation of the Protein’s Anticoagulant Activity

**Figure 2:**
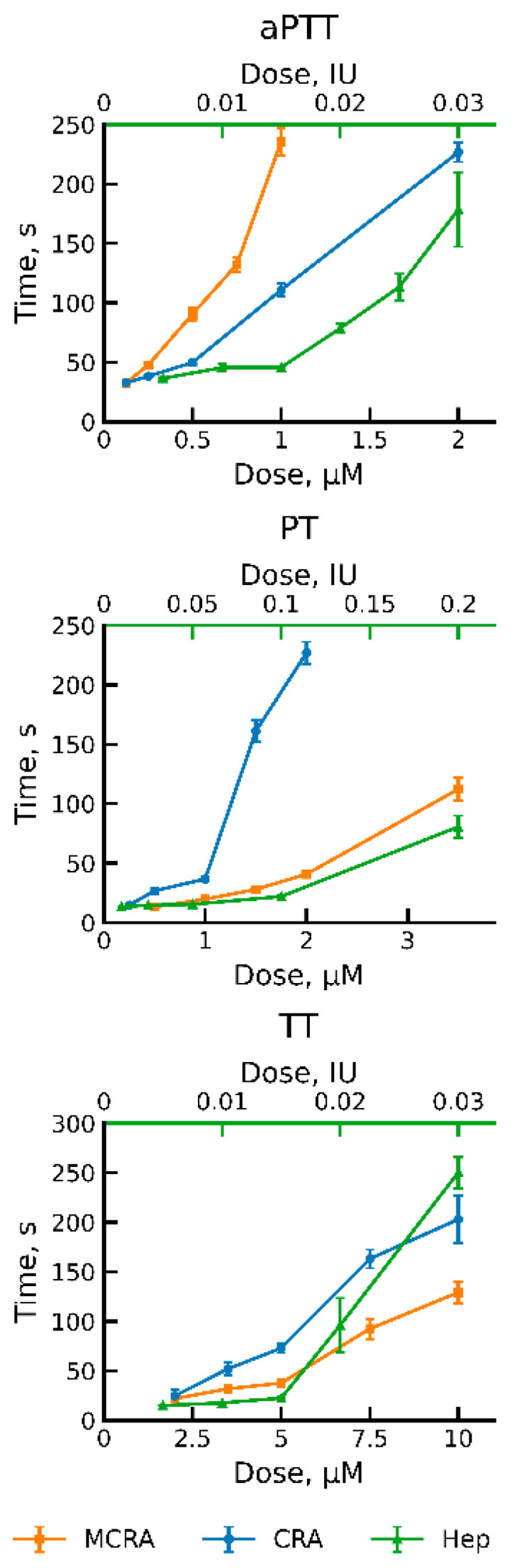
Dose–response curves showing the activity of MCRA and CRA. Clot formation times (aPTT, PT, and TT) were measured for three anticoagulants: Multiple-CRA (MCRA) and CRA, derived from the medicinal leech, and heparin (Hep). The bottom x-axis indicates the concentration of the recombinant MCRA and CRA proteins (µM). The top green x-axis shows the heparin concentration (IU). Data are presented as the mean clot formation time ± standard deviation from four independent replicates.

To assess the effect of MCRA on coagulation and compare it with CRA, we used three standard clinical tests: activated partial thromboplastin time (aPTT), prothrombin time (PT), and thrombin time (TT) [25]. As a positive control, we used commercially available medicinal heparin. It is an indirect anticoagulant of carbohydrate nature that has found the widest application in medical practice. The measurement results are presented in Figure 2. MCRA, as well as CRA, demonstrated activity in all three tests. When determining aPTT, the effective concentrations of both proteins were around 1 µM (Figure 2). However, to achieve the same time, the molar concentration of CRA had to be approximately twice that of MCRA. We suggest this is associated with the doubling of the number of domains and, consequently, functional units in the MCRA molecule. But at the same time, this effect could be related to the different specificities of the two anticoagulants. Apparently, it is the differences in specificity that lead to MCRA’s activity being weaker than that of CRA in the PT test. In the TT test, both proteins also exhibit activity, but the required concentration is an order of magnitude higher than in the previous cases. In our opinion, this indicates that both proteins possess the ability to inhibit thrombin to some extent. However, their main targets are most likely factors higher up in the blood coagulation cascade. It is possible that these two anticoagulant proteins of the medicinal leech functionally complement each other. This is indirectly evidenced by their different behaviors in the three different coagulation tests. A PTT assay reflects the efficiency of the intrinsic pathway of coagulation activation, while PT reflects the processes of activation via the extrinsic pathway. TT test is used to determine the efficiency of the final stage of coagulation—the conversion of fibrinogen to fibrin under the action of thrombin. As we can see, MCRA is more effective in the aPTT test, while CRA is more effective in the PT test. Compared to heparin, both proteins have weaker activity in the TT assay. However, the leech has a third protein, which is a very effective thrombin inhibitor, hirudin, the most well-known leech anticoagulant [26]. Thus, the medicinal leech possesses anticoagulant proteins capable of blocking coagulation activation via both the extrinsic and intrinsic pathways, as well as blocking the final stage of this process.

## 4. Discussion

In this study, we describe an anticoagulant protein that apparently arose through duplication of an ancestral gene encoding an antistasin homolog, CRA. The emergence of new paralogous genes through multiplication of ancestral structures is not a unique phenomenon; a well-known example is provided by proteins of the immune system. Among leech anticoagulants, such processes have also been described repeatedly. In a recent study, Lukas et al. [27] reported a novel protein, Tandem–Hirudin (TH), in the leech *Hirudinaria manillensis*. TH originated via duplication of the hirudin domain, accompanied by the loss of the C-terminal tail. As a result, a new protein architecture was formed on the basis of hirudin. Unfortunately, Lukas et al. were unable to identify the biological target of TH, which may reflect either a functional shift of the protein or the extraordinary difficulty of obtaining correctly folded recombinant TH.

An interesting example of multiplication and divergence among leech anticoagulant proteins was reported by Müller et al. [28]. The protein decorsin is a highly potent inhibitor of platelet aggregation. In *Haemadipsa interrupta*, the authors identified an oligomeric decorsin, Hint_DV1, containing not one but three domains. Hint_DV1 exhibited a clearly detectable ability to inhibit platelet aggregation, albeit less pronounced than expected. One might speculate that the gene encoding oligomeric decorsin represents a weakly functional genomic artifact in *H. interrupta*; however, this interpretation is most likely incorrect. The same research group subsequently identified four genes encoding oligomeric decorsins in the genome of *H. manillensis*, alongside a single gene encoding monomeric decorsin. One of these genes encodes a tetrameric decorsin, whereas the remaining three encode hexameric forms. In their later study [29], the authors elegantly proposed that expression of multimeric decorsin genes may, through alternative splicing, give rise to mRNAs encoding single-domain proteins. Indeed, recombinant hexameric decorsin Hman_DV1 was shown to lack inhibitory activity, whereas its first domain, expressed separately, was an effective inhibitor of platelet hemostasis. Monomeric decorsin from *H. manillensis* and individual domains of other multimeric decorsins also displayed inhibitory activity. These findings suggest that monomeric decorsins may indeed be generated in leeches from multimeric genes via splicing. However, an alternative explanation is also possible. The loss of activity in multimeric forms may reflect a functional shift of the protein, with the newly acquired activity remaining undetected in the assays employed. Clinical coagulation and platelet aggregation assays are performed under conditions that differ substantially from physiological ones, and novel protein functions may therefore escape experimental detection. This consideration is likewise relevant to the present study. We provide only a preliminary characterization of MCRA functionality, and important aspects may have remained unnoticed due to the limited set of methods used. Nevertheless, it can be stated with confidence that the formation of truncated MCRA forms via transcript splicing does not occur. In this study, the region encoding mature MCRA was obtained by direct PCR amplification from a cDNA library of *H. medicinalis* salivary gland cells. PCR products were cloned, and the recombinant inserts in plasmid DNA from ten independent clones were sequenced. In all cases, the inserts encoded the full four-domain form of MCRA.

The four-domain antistasin homolog MCRA is not a unique genomic artifact of *H. medicinalis.* We identified its orthologs in the transcriptomes of closely related species, *H. orientalis* and *H. verbana* [19]. Moreover, a retrospective search of GenBank revealed the presence of orthologous CRA and MCRA genes in *H. verbana* (GenBank accession numbers MT000982.1 for the gene and QPK77439.1 for the protein), annotated as antistasin-like factor E. The analyses were performed by the group of E. Macagno (San Diego, CA, USA) and the group of J.-P. Hildebrandt (Greifswald, Germany). A nearly identical MCRA transcript (98% identity) from the nerve ganglion of *H. verbana* was deposited by A. J. Northcutt et al. (GenBank: GGIQ01022094.1).

At the nucleotide sequence level, several other multimeric antistatin-like structures have also been described. A comprehensive classification study of antistatin-like proteins [2] reports antistatin homologs containing more than two domains. In oligochaetes—the ancestral group of leeches—structures with 2 to 6 domains have been identified, although their biological functions remain unknown. The same study also refers to a leech cocoon protein discovered by Manson et al. [30]. This protein exhibits a structural architecture comprising six repeats, each of which includes both an antistatin-like domain and an epidermal growth factor (EGF) domain [31]. Notably, MCRA represents the first protein-level-characterized multimeric antistatin homolog with experimentally confirmed anticoagulant activity.

## 5. Conclusions

In this study, we describe a four-domain paralog of cysteine-rich anticoagulant. The protein contains four ANS domains defined by conserved cysteine motifs; such proteins have not been described previously. Sequence comparison and phylogenetic analysis clearly support a shared evolutionary origin of the two proteins. Functional testing shows that this protein possesses pronounced anticoagulant activity and, together with CRA, affects different parts of the coagulation cascade. When combined with the action of hirudin, these anticoagulants provide *H. medicinalis* with a robust and multi-level mechanism for suppressing blood clotting during feeding.

## Figures and Tables

**Table 1:** Protein extinction coefficients.

Coefficient	MCRA	CRA
Molar extinction coefficient, M^−1^cm^−1^	36,640	19,140
Mass extinction coefficient, mg^−1^cm^−1^	1.11	1.26

## Data Availability

Data are contained within the article/Supplementary Materials.

## Supplementary Materials

The following supporting information can be downloaded at https://www.mdpi.com/article/10.3390/biom16010155/s1, Figure S1: Genetic map of plasmid pET-hmMCRA; Text S1: Nucleotide sequence coding for MCRA; Figure S2: Combined fractions of CRA and MCRA after chromatographic purification; Text S2: Amino acid sequence of the pro-MCRA; Figure S3: Multiple sequence alignment of the first subdomains of CRA and MCRA; Figure S4: Visualization of the MCRA spatial structure modeling using AlphaFold3. Supplementary Material S2: Original image of Figure S2.

## Abbreviations

The following abbreviations are used in this manuscript:

CRA	Cysteine-Rich Anticoagulant
ANS	Antistasin
MCRA	Multiple-CRA
TB	Terrific Broth
aPTT	Activated Partial Thromboplastin Time
PT	Prothrombin Time
TT	Thrombin Time
Hep	Heparin
CTM	C-terminal motif
IU	International unit
