# Supplementary material for: Duplication of the Antistasin-Like Structure Resulted in a New Anticoagulant Protein in the Medicinal Leech
Source: Biomolecules. 2026 Jan 15;16(1):155. doi: 10.3390/biom16010155 (PMC12838937; doi:10.3390/biom16010155)
Supplement: Supplementary file 1 [file biomolecules-16-00155-s001.zip › biomolecules-4056558-supplementary.pdf]

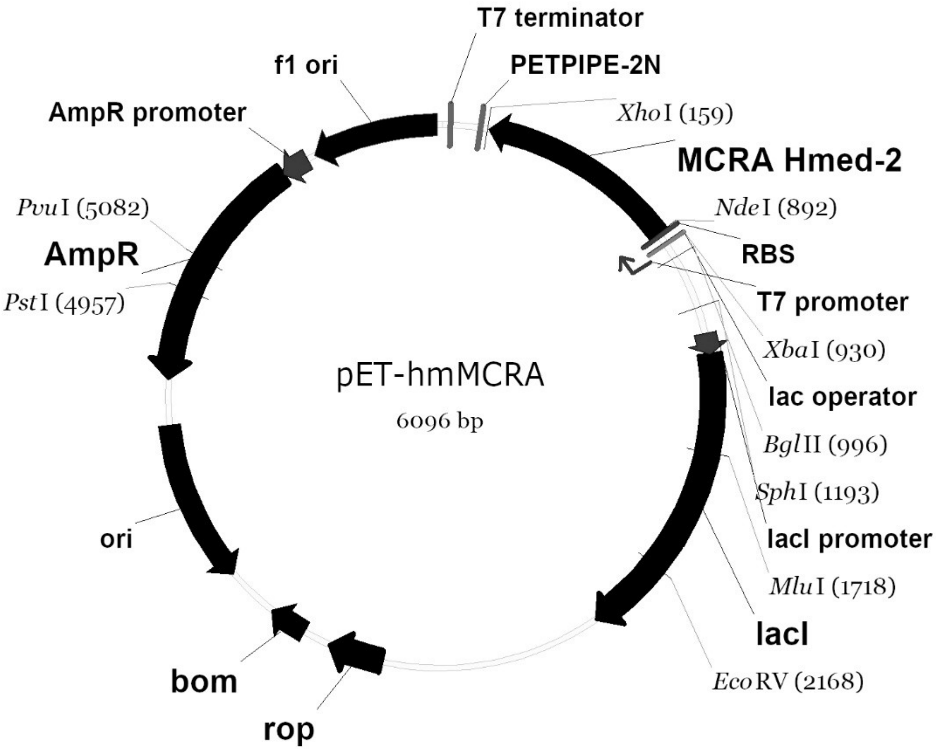

Figure S1. Genetic map of plasmid pET-hmMCRA. The recombinant plasmid pET-hmMCRA carrying the mature form of MCRA fused to a C-terminal His-Tag was constructed using the PIPE method. PCR fragments were amplified from cDNA of *Hirudo medicinalis* salivary gland cells and and pET-22b(+) vector (the pelB signal peptide sequence was omitted) and assembled without ligation into *E. coli* TOP10 cells. Colonies carrying the target plasmid were selected and verified by Sanger sequencing.

Text S1. Nucleotide sequence coding for MCRA. The sequences encoding signal peptide and stop codon are underlined.

>MCRA

ATGAAATTTGTTCGGAATATTTACTTAGTCTGTTTCCTGGTTTTCTACGCATTTAATTTTGA
AGCTGTGGCTGTACATCCTTATTTTGGCGACTATGAACTGGGATGCTTCGACGATGAATGTG
ACAATGGAAAGACTTGCAGTCCAATAACAAATGAGTGTGACTGCGATCCTGTGTACTGTCGA
ATGAATTGTGAAGTTTGGGCTAAAGACGAAAAACTTTGCGACGTGTGTGGGTGCTCGGAATC
TTGTAAGGATAGTAGGTCTTGCCGTAGAGATGAACGTTGTGATAACTTCACCAAAGTGTGTG
TTCCTCAATCATTCGAAGAAATGAAAATGTGTTGGAACAAAGGTTGCCCATGTGGTCAGCGA
TGCAACTTATATAGAAATGAATGCGAAGTAATAGCAGAGAATATTGAATGCCAAAAGGAGGA
AGAATGTCCTGATCCTTACTTATGCAGTCCTGTGACCAATCGATGTGAGTGCACTCCTGTAC
TCTGCCGAATGTACTGCAAGTTTTGGGCCAAAGACGAAAAAGGCTGCGAGATATGTAAATGT
GAAGAGCTGTGCCAGAATCAAAATTGTACTAAAGGCATGTTGTGCAGCAGCGTAACTAACAG
ATGTGATTGTCAAGACTTCAAATGTCCACAATCTTACTGTCCTCACGGATTCGAAACTGATG
AGAACGAATGCGAAGTTTGTATCTGCAAAAAACCAACTTGTGTCAACTGCGGCAAAACAACC
AAACCAAGAACTATTGACAGACTAAAAAATTGGTTCAAGAAGAAATTTGGAAAATAA

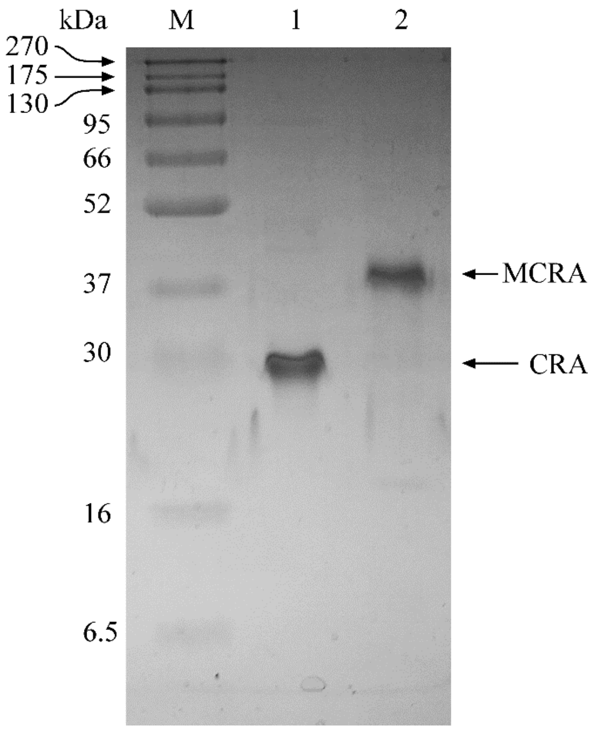

Figure S2. Combined fractions of CRA and MCRA after chromatographic purification. SDS-PAGE analysis (Laemmli) with Coomassie G-250 staining. M—protein molecular weight marker RAV-10 (Biolabmix, Novosibirsk, Russia); 1—CRA; 2—MCRA.

Text S2. Amino acid sequence of the pro-MCRA. The signal peptide sequence is underlined.

>MCRA aa

MKFVRNIYLVCFLVFYAFNFEAVAVHPYFGDYELGCFDDECDNGKTCSPITNECDCDPVYCR
MNCEVWAKDEKLCDVCGCSESCKDSRSCRRDERCDNFTKVCVPQSFEEMKMCWNKGCPCGQR
CNLYRNECEVIAENIECQKEEECPDPYLCSPVTNRCECTPVLCRMYCKFWAKDEKGCEICKC
EELCQNQNCTKGMLCSSVTNRCDCQDFKCPQSYCPHGFETDENECEVCICKKPTCVNCGKTT
KPRTIDRLKNWFKKKFGK

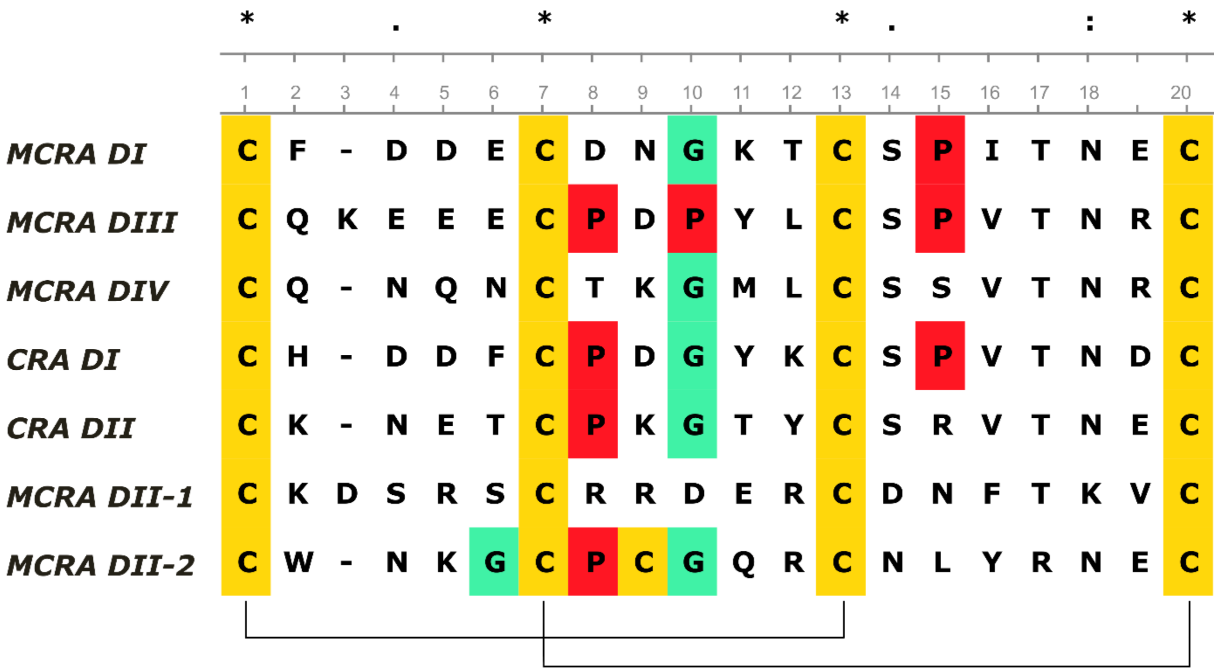

Figure S3. Multiple sequence alignment of the first subdomains of CRA and MCRA. CRA has two domains (CRA DI and CRA DII). MCRA has four domains (MCRA-D1-DIV). For the alignment, we used the sequence of each domain spanning the region between the first four cysteine residues of the antistasin motif (first subdomain). For the MCRA-DII domain, both subdomains (DII-1 and DII-2) were included. Cysteine residues are highlighted in yellow, proline in red, and glycine in green, as amino acid residues strongly influencing protein structure. Above the alignment, the symbol '*' denotes conserved positions, and the symbol ':' denotes positions with residues of similar properties. Probable disulfide bonds, located as in antistasin (PDB ID: 1SKZ [2]) are indicated Sequence analysis and alignment were carried out via the UGENE platform [22].

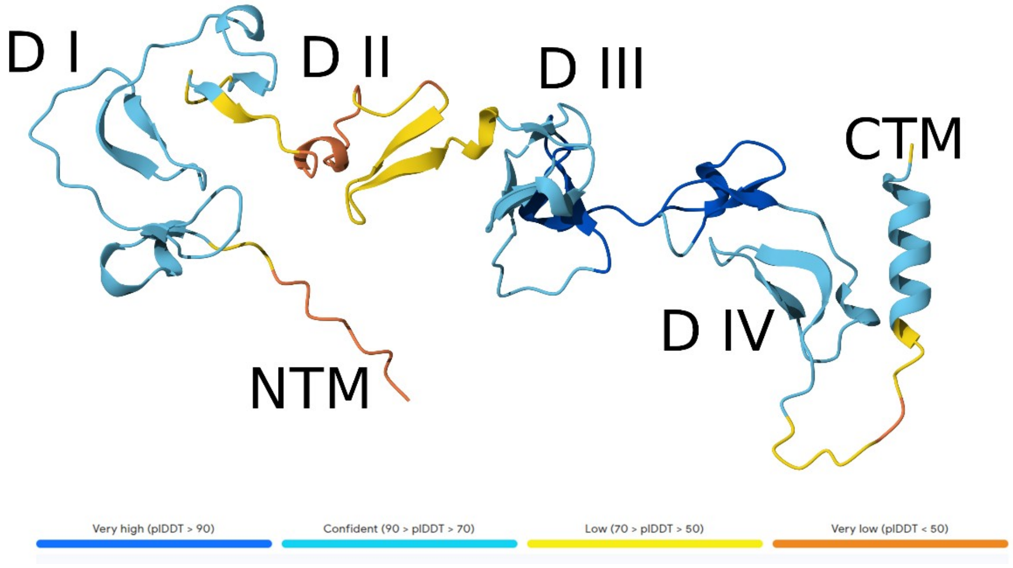

Figure S4. Visualization of the MCRA spatial structure modeling using AlphaFold3. MCRA has four well-defined domains and a separate N- and C-terminal helical motifs. Three antistasin-like domains (DI, DIII, and DIV) are clearly discernible. In contrast, the second domain (DII) adopts a different structural organization, and, as expected, the confidence level of its structural prediction is low (the color corresponds to the confidence level of its structural prediction).
