# Supplementary figures and images for: Duplication of the Antistasin-Like Structure Resulted in a New Anticoagulant Protein in the Medicinal Leech
Source: Biomolecules. 2026 Jan 15;16(1):155. doi: 10.3390/biom16010155 (PMC12838937; doi:10.3390/biom16010155)

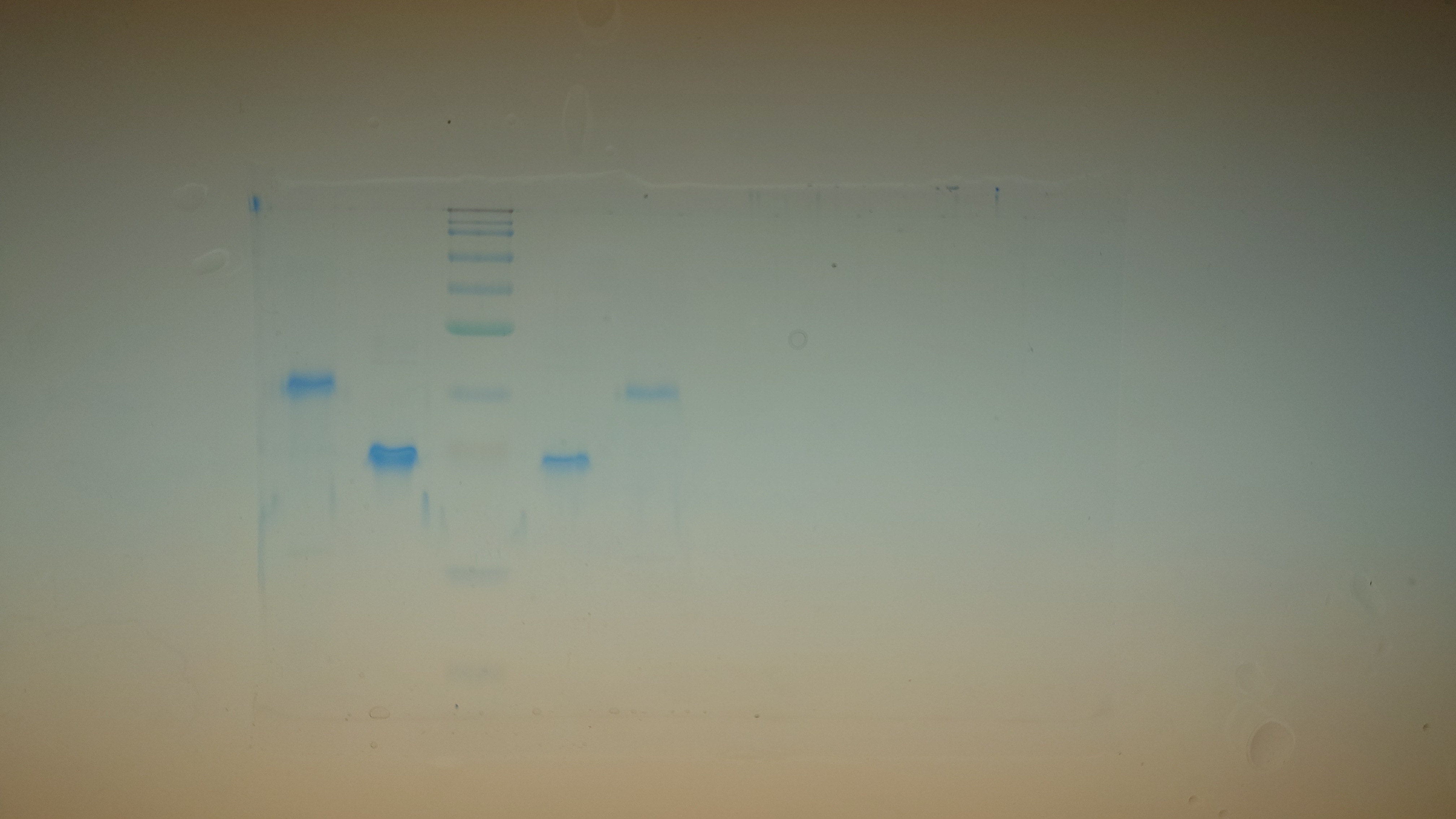

Supplement: Supplementary file 1 [file biomolecules-16-00155-s001.zip › Figure S2 raw image.jpg]
